# Supplementary material for: Are carbon emissions trading and green financial instruments synergistic? -Comprehensive quantitative research based on content analysis
Source: PLoS One. 2024 Mar 7;19(3):e0298601. doi: 10.1371/journal.pone.0298601 (PMC10919601; doi:10.1371/journal.pone.0298601)
Supplement: S1 Data — (ZIP) [file pone.0298601.s001.zip › Data/地级市Z_F工作报告.zip!/║╙▒▒/╩»╝╥╫»╩╨/└┤╫╘╣1⁄2╓┌║┼í╛┬φ┐╦╩2╛▌═°í┐_2013.docx]

各位代表：

现在，我代表市人民政府向大会作工作报告，请予审议，并请各位政协委员和列席会议的同志提出意见。

一、过去五年的工作回顾

市十二届人大一次会议以来，市政府紧紧依靠全市人民，在省委、省政府和市委的正确领导下，坚持以邓小平理论、“三个代表”重要思想、科学发展观为指导，以建设幸福石家庄为目标，围绕转型升级、跨越赶超两大任务，解放思想，开拓进取，真抓实干，圆满完成了市十二届人大一次会议确定的各项目标任务，为全面建成小康社会奠定了坚实基础。

综合经济实力显著增强。2012年，全市地区生产总值达到4500.2亿元，五年平均增长11.3%。全部财政收入完成573.2亿元，其中公共财政预算收入完成272.3亿元，分别是2007年的2.49倍和2.84倍。规模以上工业增加值完成1800.2亿元，实现利润558.6亿元，单位工业增加值利润率全国先进、全省第一。服务业增加值完成1807.3亿元，是2007年的2.06倍。自主创新能力持续增强，获省级以上科学技术奖122项。县域经济发展加快，全部财政收入超10亿元的县（市）由3个增加到7个，其中超20亿元的达到2个，突破百亿元的1个。淘汰落后产能任务超额完成，节能减排目标如期实现。

重点项目投资快速增长。2012年，固定资产投资完成3673.3亿元，五年平均增长26.7%。五年累计竣工省市重点项目287个，新开工项目533个，格力电器、欣意电缆等一批省市重点项目竣工投产。石家庄经济技术开发区被批准为国家级开发区，全市所有的县（市）都建成了省级以上工业聚集区或经济技术开发区。石太客专、京石武高铁建成通车；京昆高速（石保段）、西柏坡高速、三环公路等重大交通工程建成投用。石家庄国际机场改扩建主体完工。南水北调京石段建成通水。

省会面貌发生明显变化。围绕城镇建设“三年大变样、三年上水平”，高起点完成城市总体规划和各类专项规划，实现控制性详规全覆盖，形成了“一河两岸三组团”省会发展新格局。旧城改造全面展开，城市建设管理水平明显提升，市容环境大为改观，累计新增公园广场20座、街心游园112座，建成区绿地率达到41%。铁路穿城入地工程基本完工，新客站顺利投入使用；城市轨道交通开工建设；“公交都市”建设步伐加快，新开辟公交线路122条，公交分担率达到31.1%。正定新区路网建设全面铺开。强力实施蓝天碧水、洨河治理和市容市貌综合整治十大工程，环城水系全线通水,滹沱河综合整治一期工程基本完工。拆改市区分散燃煤锅炉974台，关停治理建材企业546家，累计淘汰黄标车6.6万辆。县城和小城镇建设步伐加快，全市城镇化率由45.1%提高到53.5%。

“三农”工作取得可喜成绩。各级财政用于“三农”的支出五年累计达到350亿元，年均增长34.5%。农业综合生产能力稳步提高，粮食生产实现“九连丰”。农业产业化步伐加快，市级以上重点龙头企业达到255家，农业产业化经营总量达到874亿元。畜牧、蔬菜、林果等优势产业规模不断壮大。农业科技推广效果显著，小麦玉米良种覆盖率达到98%以上。农村基础设施建设持续加强，五年累计新建改造农村公路5567公里，完成37座水库除险加固，新建改造变电站100余座，改造中低产田47万亩，发展节水灌溉168万亩，解决了207.6万人的饮水安全问题。启动建设省级新民居示范村740个，省级幸福乡村示范点达到176个。太行山绿化、环省会生态建设取得实效，森林覆盖率达到32.2%。

改革开放迈出坚实步伐。105家国有和集体企业、6家经营性事业单位、80个城中村集体经济组织完成改制，北人集团、宝石集团等一批大中型企业成功重组。投融资体制改革取得新进展，市场化融资平台不断完善，五年新增上市公司17家，小额贷款公司54家。对外开放成效明显，成功举办了石洽会、正博会、动博会、药博会等一系列投资促进和商贸会展活动，沃尔玛、家乐福、爱立信等世界500强企业前来我市投资置业。与31家央企展开合作，引领带动作用明显提升。五年累计实际利用外资34亿美元，引进市外资金1710.8亿元；外贸进出口总额累计完成512亿美元，年均增长21.4%。

城乡居民生活持续改善。全市财政用于民生的支出五年累计达到1188亿元，占财政总支出的比重达到73.9%。2012年，城市居民人均可支配收入达到23038元，五年平均增长11.8%；农民人均纯收入达到8993元，五年平均增长12.7%。五年城镇新增就业48万人，转移农村劳动力73.9万人，15.3万农村贫困人口实现稳定脱贫。城镇登记失业率控制在4%以内。山区教育扶贫工程成效显著，建设寄宿制学校56所，3.1万名山区学生实现集中就读；义务教育均衡发展水平进一步提升。市图书馆、美术馆、广电中心等标志性文化设施相继建成，四级公共文化服务网络基本形成。精神文明创建活动不断深入，“善行河北”主题道德实践活动广泛开展，文化惠民活动丰富多彩，一批原创精品力作获国家级大奖。城乡居民社会养老保险全面推开，城乡居民医疗保险向全覆盖迈进。重度残疾人特殊保障政策在全省率先实施。政府办社区卫生服务机构、乡镇卫生院、村卫生室实行了基本药物零差价销售，全面完成村卫生室标准化建设。全市保障性安居住房累计开工建设11.7万套，发放廉租住房补贴2.13亿元。全民健身运动广泛开展，竞技体育取得好成绩。认真落实计划生育基本国策，保持了稳定的低生育水平。社会救助和养老服务体系进一步完善。援藏、援疆、援建四川平武任务圆满完成。国防动员和双拥共建深入开展，连续七次被评为“全国双拥模范城”。妇女、儿童、老龄、残疾人、慈善等各项事业不断发展，民族宗教、外事侨务、防震减灾、气象、人防、档案等各项工作都取得新成绩。

政府自身建设不断加强。不断深化行政管理体制和政府机构改革，政府职能进一步转变。坚持依法行政，自觉接受人大法律监督和工作监督、政协民主监督和社会监督，累计办理人大代表建议1675件、政协委员提案2602件，办结率均达100%。大力深化行政审批制度改革，五年累计削减行政许可项目136项，削减率达53.5%。社会管理创新综合试点工作扎实推进，110座综合警务服务站建成投用，社会治安综合治理不断加强。圆满完成北京奥运会、建国60周年大庆、党的十八大等重大活动安保工作。全市政治稳定，社会安定，人民群众安居乐业。

各位代表，五年成绩的取得，是省委、省政府和市委正确领导的结果，是全市人民团结奋斗、共同努力的结果。在此，我代表市人民政府，向全市人民，向人大代表、政协委员，向各民主党派、工商联、无党派人士、人民团体和社会各界人士，向驻石人民解放军、武警官兵，向中直、省直机关驻石各单位，向所有关心石家庄发展的国内外朋友，致以崇高的敬意和衷心的感谢！

在肯定成绩的同时，我们也必须看到，我市经济和社会发展中还面临着一些困难和问题，主要是：统筹城乡发展任务艰巨，城镇化进程不够快，县域经济实力还比较弱；产业结构不尽合理，新兴产业、高新技术产业比重偏低，服务业发展相对滞后；科技创新能力不强，节能减排任务繁重，群众关注的大气污染、交通拥堵等问题还比较突出；经济增长下行压力和产能相对过剩的矛盾依然存在；政府职能转变还不到位，发展环境需要进一步改善。对此，我们必须保持清醒认识，进一步增强忧患意识，强化危机意识，以对全市人民高度负责的精神，采取有力措施，切实加以解决。

各位代表，回顾五年来不平凡的历程，我们深切感到，做好新形势下的政府工作，必须坚持以科学发展观为指导，围绕转型升级、跨越赶超、建设幸福石家庄的总目标，大力推动中东西三大区域协调发展，大力实施工业强市战略，大力改善生态环境，努力走出一条具有石家庄特色的发展新路子；必须坚持抓项目就是抓发展、抓大项目就是抓大发展、抓一批大项目就是抓跨越发展的理念，努力扩大经济总量，提高发展质量；必须坚持深化改革，扩大开放，加快形成有利于转变发展方式的体制机制，努力增强发展的活力和动力；必须坚持以人为本，高度关注民生，努力激发广大人民群众投身建设和发展的积极性；必须坚持优化发展环境，大力推进效能革命，努力营造风清气正、干事创业的浓厚氛围。这些经验来之不易，十分宝贵，我们必须倍加珍惜，在继承中发展，在实践中创新，引领我们不断取得新的胜利！

二、今后五年的目标任务

站在新的起点，我市面临前所未有的发展机遇：党的十八大明确提出了全面建成小康社会的宏伟目标；国家实施首都经济圈发展规划和京津冀一体化发展战略；市第九次党代会描绘了我市未来发展宏伟蓝图；我市经济和社会事业进入快速健康发展新阶段。这些难得机遇和有利条件，必将凝聚起全市人民强大的思想共识，激发出强大的精神力量。只要我们解放思想，真抓实干，开拓进取，就一定能够赢得主动，乘势而上，开创全市率先发展、创新发展、跨越发展的崭新局面！

今后五年政府工作的总体要求是，坚持以邓小平理论、“三个代表”重要思想、科学发展观为指导，全面贯彻落实党的十八大、省第八次党代会、市第九次党代会精神，牢牢抓住新一轮发展机遇，积极融入首都经济圈，深入实施中东西三大区域协调发展战略，紧紧围绕主题主线，深化改革开放，强化创新驱动，优化发展环境，改善生态环境，保障改善民生，维护社会稳定，促进新型工业化、新型城镇化、信息化和农业现代化同步发展，全力打造现代一流省会城市，加快转型升级、跨越赶超、建设幸福石家庄步伐，确保在全省率先全面建成小康社会。

今后五年，全市经济社会发展的总体目标是：全面完成“十二五”规划和市第九次党代会提出的目标任务。地区生产总值年均增长10%以上，比全省提前一年实现翻番。全部财政收入年均增长13%以上，其中公共财政预算收入年均增长14%以上。城乡居民收入年均分别增长11%以上。全市经济增长的质量和效益大幅提升，综合经济实力在全省领先，主要发展指标在全国省会城市中位次前移。

围绕实现上述奋斗目标，必须在以下方面迈出更大步伐、取得更大成效。

第一，提升综合经济实力，打造京津冀第三极。抓住国家实施京津冀一体化发展战略的机遇，主动承接京津地区产业转移和要素辐射，加强与京津地区的交通互联、产业互补、园区共建、资源共享，不断拓展融合发展的深度和广度。推进经济结构战略性调整，大力发展生物医药、高端装备制造、电子信息等新兴产业；加快发展现代物流、服务外包、总部经济、金融保险、旅游会展等现代服务业，促进服务业超常发展、高端发展，占生产总值的比重达到50%。大力发展现代农业，持续增强农业综合生产能力，加快构建新型农业经营体系，推动农业大市向农业强市转变。实施创新驱动发展战略，建设国家创新型城市。

第二，加速城镇化步伐，建设现代一流省会城市。按照“一河两岸三组团”省会发展新格局，全面提升城市规划、建设、管理水平。强化规划的刚性约束，以高水平的规划设计，引领城市建设出品位。全面加强主城区基础设施建设，加快城市地铁建设进程，推进高铁、航空和高速公路、干线公路、快速公交等交通设施建设，不断提升省会的交通枢纽功能；加快改造主城区水、电、气、热等市政公共设施，全面提高保障能力。高标准规划建设环城水系两岸区域，推动主城区外延发展，拓展城市空间。按照“低碳、生态、智慧”的理念，大力推进正定新区建设，加快完善道路和生产生活配套设施，提升新区聚集产业和吸纳人口的能力，尽快使30平方公里核心起步区形成规模。提升城市精细化管理水平，创新城市管理体制，实施智慧城市建设工程，努力形成高效快捷的城市管理系统。提速县城和特色镇建设，促进县城扩容升级，支持辛集、平山、晋州、新乐等县（市）向中等城市迈进。大力推进农村城镇化建设，深入实施幸福乡村计划，力争农村新民居建设示范村达到1000个以上，幸福乡村示范点达到500个以上；加快户籍制度改革，有序推进农业转移人口市民化，积极探索具有石家庄特色的新型城镇化之路，全市城镇化率达到60%以上。

第三，发挥园区优势，建成具有重大支撑作用的产业基地。集中力量做大做强现代产业基地，确保主要经济指标增速高于全省平均水平。推动高新技术开发区率先发展，力争主营业务收入突破4000亿元，在国家级高新区综合排名中位次前移。促进经济技术开发区、循环化工园区快速发展，实现主营业务收入2000亿元和1500亿元以上。力促装备制造基地、生物医药基地、信息产业和半导体光谷基地主营业务收入跃上新台阶。

第四，改善生态环境，建设绿色宜居家园。坚持生态立市，推进绿色发展。继续大力实施“蓝天碧水”工程，完成主城区全部重点污染企业的搬迁改造；加强大气污染治理，重点开展燃煤污染、扬尘污染、水泥建材行业污染综合整治。加强水环境治理，严格水源地保护，实现辖区主要河流和企业的自动化监控，确保污水处理达标排放。探索建立生态补偿机制，加大西部山区生态屏障建设力度。大力度推进环省会绿化工程建设，大规模开展植树造林活动，力争全市森林覆盖率每年净增1个百分点以上。

各位代表，年初的雾霾天气警示我们：生态环境已经到了非抓不可的时候了，我们必须站在抓生态环境就是抓发展、就是抓民生的高度，以壮士断腕的勇气，切实加大环境污染治理力度，经过五年坚持不懈的努力，实现全市大气和水环境明显改善，让全市人民都能呼吸洁净的空气、喝上放心的水！

第五，实施“腾飞计划”，推动县域经济跨越发展。按照中东西三大区域发展的战略定位，强力推进中部率先隆起、东部整体突破、西部加快发展。重点提升县域工业整体水平，培育和扶持一批主业突出、特色鲜明、体系完整、环境友好的龙头企业。有序推动省会主城区产业向县域扩张，基础设施建设、重大项目布局向县域倾斜，资金、技术、人才向县域聚集，大力推进47个县域产业集群提档升级，力争营业收入50亿元以上的产业集群达到30个、100亿元以上的达到15个以上，实现县域经济转型发展、总量倍增。

第六，建设文化强市，提升文化软实力。坚持社会主义先进文化前进方向，推进文化事业大繁荣、文化产业大发展。深化公民道德建设，广泛开展“善行河北”主题道德实践活动，提升全社会文明程度，努力争创全国文明城市。深入开展文化惠民活动，健全公共文化服务体系，保障人民群众的基本文化需求。关心和培养文化名家和领军人才，持续推出具有石家庄特色、体现时代精神的精品力作。围绕做大做强现代传媒、动漫创意、民俗文化等六大文化产业，培育一批文化强企，引进一批战略投资者，建设一批重大文化项目。加强文物保护，加大对文化资源的挖掘整理和传承创新，努力打造正定古城文化品牌、“大西柏坡”红色文化品牌、反映时代风貌的现代文化品牌，不断增强省会文化底蕴。

第七，保障改善民生，全面建设幸福石家庄。坚持以人为本、民生为重，努力在学有所教、劳有所得、病有所医、老有所养、住有所居上取得重要进展。优先发展教育事业，全面推进教育综合改革，努力办好人民满意的教育。加快发展医疗卫生事业，加大医疗投入，改善医疗环境，提高医疗质量。实施更加积极的就业政策，不断提高就业的质量和稳定性。建立市场配置和政府保障相结合的住房制度，逐步缓解低收入群体的住房困难。制定实施城乡居民收入倍增计划，努力提高城乡居民收入。健全城乡社会保障体系，不断提高统筹层次和保障水平，切实做到应保尽保。建立政府主导和社会参与、居家养老和机构养老相结合的养老服务体系。继续抓好社会管理创新综合试点市建设，加快形成源头治理、动态管理、应急处置相结合的社会管理机制。强化市场价格监督，保持物价基本稳定。

各位代表，新蓝图令人鼓舞，新目标催人奋进。让我们团结一心，埋头苦干，奋力争先，努力创造无愧于时代、无愧于人民、无愧于省会称号的辉煌业绩！

三、2013年的重点工作

今年是全面贯彻落实党的十八大和全国“两会”精神的第一年，是实施“十二五”规划承前启后的关键一年。做好今年工作，意义十分重大，必须牢牢把握稳中求进的工作总基调，紧紧围绕提高经济增长质量和效益这个中心，把稳增长、调结构、惠民生、优环境作为基本着力点，努力实现各项工作新突破。综合考虑各方面因素，今年全市经济社会发展的主要预期目标是：地区生产总值增长10%以上，全部财政收入增长15%，其中公共财政预算收入增长18%；规模以上工业增加值增长13%；固定资产投资增长20%，社会消费品零售总额增长15%以上；单位生产总值能耗下降3.89%；城市居民人均可支配收入增长11%，农民人均纯收入增长11%；城镇登记失业率控制在4.5%以内。围绕实现上述目标，重点抓好八个方面工作：

（一）全力以赴，加快重点项目建设。把上项目、增投入、扩总量，作为推动经济社会持续快速健康发展的第一选择，突出重点，强化责任，全力推进。

加快重大产业项目建设。对今年安排的125个省市重点项目，优先保障土地、资金等要素供应，确保南车装备制造、通用航空二期、纵横光缆产业基地等71个项目开工建设；加快辛化搬迁升级、智能化电力设备产业园等46个续建项目建设进度；加大浙商机电产业园等8个前期项目跑办力度。年内确保石炼化800万吨油品质量升级改造、中友机电等83个项目竣工投用，石炼化百万吨轻烃综合利用完成立项。对已签约的27个外资项目和35个央企合作项目，逐一制定专项推进计划，确保早开工、快建设。

全力推进产业园区提档升级。制定专项扶持和激励办法，力促省级以上产业园区基础设施大提升、发展环境大改善、优势产业大发展。不断提高高新技术开发区、经济技术开发区、循环化工园区、装备制造基地、信息产业基地、南部工业区等重点园区的配套能力，率先完成路、水、电、讯、气、热等基础设施建设。强化省级经济技术开发区、产业聚集区的载体功能，发挥比较优势，打造园区特色，拉长产业链条，提高园区的整体实力和竞争力。加快华药总部基地及研发中心、中国服装创意产业园等现代产业园区的推进力度，力争年内开工建设。

加大重大项目谋划和招商力度。围绕我市主导产业、优势产业和战略性新兴产业，加强项目谋划包装，广泛开展招商引资，确保做到谋划一批、签约一批、建设一批、储备一批，保持项目建设强大后劲。年内，藁城、鹿泉、正定、栾城四个县（市）分别新建2个10亿元以上项目，其它县（市）区分别新建2个5亿元以上项目。市级重点项目储备稳定在1000项以上。

（二）抓住关键，推进工业转型升级。按照做实对标、做强龙头、做活民营的要求，强力实施工业突破，大力提高工业的整体水平。

继续深化拓展对标行动。引导各个行业和企业，对标国际、国内一流水平，在研发、生产、销售、管理等环节实施全过程对标，制订追赶、超越、创标路线图，通过技术创新和技术改造，全面提升企业的质量和效益，降低单位增加值能耗和排放。年内争创国内标杆指标200项，培育对标示范企业80家。进一步加大钢铁、热电、纺织服装、医药化工等传统产业改造提升力度，重点抓好石煤机矿山机械研发制造中心、威远生化石家庄基地等15个传统产业升级改造项目建设，滚动实施市重点技改项目200项以上，全年技改投资力争超过1000亿元。

大力发展战略性新兴产业。优化政策环境，激发市场主体活力，促进生物医药、高端装备制造、信息技术等新兴产业突破瓶颈制约，总量快速扩张，实力大幅提升。年内重点抓好光谷二期、旭新光电、通用航空等57个重大新兴产业项目建设，全市规模以上战略性新兴产业增加值增长22%以上。

做大做强龙头企业。以50家骨干企业和高成长型企业为龙头，大力实施“跨五超百冲千”工程，鼓励企业兼并重组，推动生产要素向优势企业和产品集聚，加快培育一批国际国内知名品牌，打造一批主营业务收入跨50亿、超百亿、冲千亿的“企业航母”。年内，力争阳煤正元等5家企业主营业务收入超50亿元，华药、石药等7家企业超百亿元。

提高科技创新水平。进一步强化企业创新的主体地位，年内新认定高新技术企业25家。积极推进与北京大学、清华大学等高等院所的合作，充分发挥省会高校、科研院所的科技资源优势，加快建立产、学、研相结合的创新合作机制。拓展以石药、华药等优势企业为主体的产学研联盟，支持有条件的企业建立研发平台，力争健海生物芯片工程实验室等7个高技术研发平台投入使用。加快创新研发体系建设，建立科技创新公共服务平台，新培育市级以上企业技术中心、工程(技术)研究中心和实验室15家。围绕我市重点产业，抓好重大科技专项实施，力争取得国内先进水平以上科技成果170项。认真落实国家知识产权战略，进一步加大知识产权保护力度。加强创新人才队伍建设，大力营造尊重知识、尊重人才、尊重创造的浓厚氛围，以高素质人才引领高水平发展。

切实抓好节能减排。积极推进低碳城市试点建设，加快发展循环经济和节能环保产业。突出抓好新老“双三十”、“双千”企业治理，组织实施节能技改项目100项，完成节能50万吨标准煤。降低重点行业和企业的能耗和排放总量，坚决改造淘汰落后产能，确保完成年度节能减排目标任务。大力度推广绿色节能建筑，鼓励开展建筑垃圾、生活垃圾再利用，促进经济社会可持续发展。

（三）创新方式，力促服务业发展提速。把加快发展服务业作为优化产业结构、促进经济发展的最大潜力和增长点，培育服务业支撑项目，发展新的消费业态，力促现代服务业超常发展。

做大做强生产性服务业。加快发展现代物流业，提升南部综合、正定商贸两大省级物流聚集区，确保润丰物流、洛杉奇冷链物流园等8个项目建成投用，润德物流园等9个项目开工建设；大力度推进空港工业园建设，年内力争综合保税区获得国家审批。加快石家庄国际服务外包开发区建设，努力培育新的经济增长点。立足省会资源优势，大力发展楼宇经济、电子商务、研发设计、文化创意等新兴服务业。积极发展金融保险业，重点抓好省会金融核心区、金融后台服务中心建设，争取更多的国内外各类金融机构落户我市。积极推进三网融合试点工作，实施新一代互联网、物联网应用示范工程。

活跃提升生活性服务业。按照做强核心商业区、优化二级商业区、延伸社区商业网点的思路，合理规划省会商业布局，促进商业持续健康发展。抓好市区40个重点商贸项目和城市综合体建设，力争中储红星美凯龙等10个项目开工建设，确保华强广场等11个项目竣工开业。大力支持北国、万达、东购、勒泰、万象天成等一批重量级商贸企业做大做强。加快推进新华集贸、南三条市场改造升级。建立中小商贸企业公共服务平台，支持中小商贸企业加快发展。年内每个县（市）启动建设1个大型物流中心和1个商贸中心。加快发展以家政服务、社区服务、养老服务、疾残护理为重点的家庭服务业。

倾力打造服务业品牌。进一步提升药博会、正博会等商贸会展活动水平，推动会展经济加快发展。发挥高铁和航空的交通优势，加大旅游资源规划开发和市场开拓力度，叫响大西柏坡红色旅游名片，提升赵州桥、苍岩山、嶂石岩等十大景区形象，推进驼梁——五岳寨——天桂山景区整体升级，加大段家楼景区的保护和开发力度，力争更多景区进入5A行列。旅游业总收入增长20%以上。

努力扩大居民消费。改善消费环境，丰富经营形式，提高消费能力。大力发展信用消费、网络购物等新型消费业态，加快发展电子商务、连锁经营、物流配送等现代流通方式。建设国家电子商务示范基地，力争引进国内外知名电子商务企业20家以上。集中力量推进夜经济工程建设，继续鼓励和支持各商家延长营业时间，活跃经营方式，不断提升夜经济的档次、品位和规模。深入推进“万村千乡”市场工程、新网工程和“双进工程”，加强农村商贸中心和配送中心建设，认真落实新“国五条”房地产调控政策，促进房地产业平稳健康发展。

（四）多措并举，统筹抓好“三农”工作。坚持把“三农”工作放在重中之重位置，构建集约化、专业化、组织化、社会化相结合的新型农业经营体系，加快农业现代化和农村小康建设步伐。

提升农业综合生产能力。在稳定880万亩耕地保有量基础上，重点抓好500万亩粮食生产核心区建设，总产稳定在510万吨以上。大力发展设施农业、都市农业，提高农业生产综合效益。发展壮大畜牧、蔬菜、林果等优势产业，年内创建标准化规模养殖示范场150个、标准化蔬菜示范园20个。加大农业科技创新和技术推广力度，建立健全农业社会化、信息化服务保障体系。

提高农业产业化水平。坚持以工业化理念发展农业，支持农业公司、农业合作社加快发展，鼓励广大农民以多种形式入社、入公司经营，提高农民的组织化程度。按照依法、自愿、有偿的原则，积极吸引社会资本注入农业，引导农村土地承包经营权有序流转，鼓励和支持承包土地向专业大户、农业合作社集中，发展多种形式的适度规模经营。抓好市级以上龙头企业、省级农产品加工示范基地县建设，加快推进190个农业产业化项目，力争农产品加工转化率达到76%以上。

改善农村生产生活条件。按照“水电路讯房、山水林田村、科教文卫保”十五字方针，加强农村基础设施建设，新增农村公路445公里，治理中小河流3条，除险加固水库20座，治理水土流失160平方公里，发展节水灌溉53万亩。深入开展农村环境综合整治，加强农村“空心村”治理，完成1293个重点村庄“四清四化”和“六改两建”。每个县（市）区创建2个市级以上生态镇、10个卫生村。新民居建成区全部实现道路硬化、集中供水供暖、生活垃圾和污水无害化处理。

促进农民增加收入。继续实施农民增收帮扶工程，扎实开展基层建设年活动，努力在政策、技术、培训、信息、劳务输出等方面提供支持和服务，促进农村劳动力有序转移，大力增加农民工资性收入，确保农民收入增长高于城镇居民收入增长。集中力量打好扶贫攻坚战，切实抓好264个扶贫开发重点村整村推进计划，加大对国家扶贫工作重点县的支持力度，确保4万人实现稳定脱贫。

（五）对标先进，推进城镇建设上水平。以天津、成都市为标杆，围绕打造“河北第一窗口”，完善配套设施、提升城市功能、聚集优势产业、强化精细管理，大力实施“四大工程”。

新区建设提速工程。完成新城大道、太行大街建设，形成主城区与正定新区快速通道。加快市民服务中心建设，力争年底前具备入驻条件；加快会展中心、河北奥林匹克体育中心、职教园区、市一中新区分校、市特殊教育职业中专学校、河北医科大学第二医院新区医院、市第一医院新区分院、河北出版集团等项目建设进度；加快实施正定古城保护，启动古城墙修复工程，恢复一批古街巷历史风貌；加快完善新区路网、公建配套项目，全面推进新区各项建设，充分展示新区的美好形象。

基础设施完善工程。启动石济客专建设，推进京昆高速(石太段)、京港澳高速（京石段、石安段）、107国道、定魏公路、平涉线等改扩建工程，完成晋深公路等5条干线公路建设。启动南二环东延、西延工程，确保红旗大街南延工程、三环辅路、机场路建成投用。进一步完善市区路网，开工建设新胜利大街、南二环与新胜利大街立交桥等5项配套工程。高标准完成新客站东广场建设。稳步推进旧城和城中村改造。全力推进城市轨道交通项目建设，确保年内完成1号和3号线一期工程各站点主体工程量的20%。加快“公交都市”建设，启动白佛、汇通路、中仰陵等5个公交枢纽站建设，完成平安大街、建设大街、体育大街3条公交专用道建设和延伸工程，公交分担率达到32.6%。加快热源和管网建设步伐，保障城区供热质量。加大市区排水管网改造力度，推进民心河二期、五支渠、东二环地下管网南延等雨污管网工程建设，提升城市防汛能力。

城市精细化管理工程。创新城市管理体制，坚持建管并重、综合治理，构建管理与执法高效统一的大城管格局。加快智慧城市建设，整合规划、城管、交通等信息资源，构建信息互通、资源共享平台。深入实施城市容貌综合整治工程，以新旧火车站片区、二环路、平安大街等“三片、一环、八线”为重点，加大对违法违规建筑的监督和查处力度，实施净化、美化、亮化、绿化、硬化。管住管好建筑渣土、工地扬尘和噪音污染，进一步提升道路保洁水平，二环内主干道机械化清扫率达到80%以上。

县城扩容升级工程。按照突出特色、彰显个性、完善功能、打造精品的要求，加快推进路网、水网、气网、热网、电网等配套设施建设，大力提高县城综合承载能力。年内每个县（市）至少建成1条以上标志性街道，1个面积不低于3万平方米的公园，2块面积不低于5000平方米的绿地。大力推进县城周边一公里范围内的村庄向县城集聚。

（六）深化改革开放，增强科学发展新动力。坚持把深化改革扩大开放，作为加快发展的最大“红利”，充分激发各类市场主体的活力和动力。

继续深化重点领域改革。积极推进行政管理体制和事业单位分类改革，稳妥推进经营性事业单位产权制度改革。深化财政体制改革，加快建立基本公共服务均等化的公共财政体系。全面开展农村土地确权登记，稳步推进农村集体产权制度改革。深化收入分配制度改革，不断优化收入分配结构。加大国有企业优化重组力度，支持北人集团、物产集团等4家企业实施兼并扩张，完成东方热电等15家国有企业改制重组。扶持企业上市直接融资，年内力争汇金机电、科林电器等企业成功上市。

全方位扩大对外开放。坚持内资外资并重，引资引智并举，不断拓展对外开放的深度和广度。精心办好石洽会等重大经贸活动，有针对性地办好新一代电子信息、高端装备制造、创新创意创业等产业招商会。主动对接世界500强和行业领军企业，积极参与百家央企、百家院所高校和百家知名民企进河北活动，力争中化集团、美国普洛斯集团等战略投资者到我市投资置业。创新招商方式，面向国内外广泛开展招商活动，扩大与京津、长三角、珠三角等重点地区的协作。充分发挥海外华侨华人作用，大力引进高新技术合作项目，促进“引资、引智、引技”有机融合。坚持出口和进口并重，力争全年外贸进出口总值增长3%。全年实际利用外资达到9亿美元，引进市外资金800亿元。

发展壮大民营经济。全面落实鼓励民营企业发展“新36条”和省、市支持非公有制经济发展若干政策，毫不动摇支持非公有制经济发展。加强融资担保、创业辅导、政策培训等服务平台建设，激发全民创业活力。加大金融创新力度，支持各县（市）建设和提升投融资平台，努力拓宽融资渠道。加大对中小企业的支持力度，年内确保新增规模以上工业企业200家以上。扶持民营科技企业和小微企业健康成长，努力扩大市场主体数量，加速形成一支数量庞大的企业群体。年内全市民营经济增加值、上缴税金分别增长15%和17%以上。

（七）攻坚克难，持续改善生态环境。围绕打造舒适宜居的生态环境，坚持铁腕治污，刚性降耗，强力推进三大攻坚行动。

实施大气环境改善攻坚行动。深入开展重点行业和企业、机动车尾气、扬尘烟尘、分散燃煤、油气挥发等十大污染综合治理工程，突出抓好钢铁、热电、水泥建材等重点行业集中治理，完成7家电厂除尘设施升级改造，实施火电行业、重点钢铁和水泥企业脱硫脱硝工程；关、停、并、转、改多措并举，完成125家企业粉尘污染治理；搬迁改造2家市区高排放企业。对重点污染企业，实施强制性清洁生产审核制度。对新建、改建、扩建项目，严格进行环境影响评价。加快市区分散燃煤锅炉、燃煤大灶拆改治理，集中整治建筑工地和道路扬尘、机动车尾气超标、农作物秸秆焚烧污染。加快建设8个大气污染控制示范区，实现粉尘治理、绿化硬化、排放治理“三个全覆盖”和环境卫生无死角。加强PM2.5、PM10、二氧化硫、氮氧化物等污染物的监测治理，最大程度改善空气质量。

实施水环境综合整治攻坚行动。以主要河流水污染防治和饮用水水源地保护为重点，集中实施“八大工程”。清理岗南、黄壁庄水库饮用水源保护区内的污染物，整治水库上游企业，封堵保护区内所有排污口，确保集中式饮用水源地水质全部达标。开展“三河两渠一沟”（滹沱河、洨河、磁河,石津灌渠、邵村排干渠,汪洋沟）等重点河流的综合治理，建立全天候电子监控系统和水质自动监测站，确保监测断面水质达到省考核标准。继续大力度推进洨河综合整治，全面完成桥东污水处理厂扩建升级等20项工程，确保6月底实现洨河碧水清流。

实施绿色生态建设攻坚行动。全市新增造林绿地57万亩以上。全面推进环省会绿化工程建设，抓好高速公路、国省干道两侧等重点区域植树绿化，建设绿色走廊；抓好西山森林公园、三环路绿化提升、滹沱河百里绿色长廊、环城水系沿线绿化、太行山绿化工程建设。全面提升主城区园林绿化水平，建成二环路景观“绿环”，启动新胜利大街9公里“中央绿轴”工程，实施学府路、友谊大街等5条道路绿化工程，对建设大街、谈固大街等“6纵”主干道进行绿化提升改造，建成20块街旁游园绿地。主城区新增乔木300万株，新增绿地600万平方米。在全市农村广泛开展“四旁”植树活动，全市森林覆盖率达到33%以上。

（八）保障民生，全面加强社会建设。加大公共资源优化配置力度，大力促进公共服务均等化，努力让更多发展成果惠及全市人民。

加快发展文化教育事业。继续推进义务教育均衡发展，确保7个县（市）区通过义务教育基本均衡评估验收；改造提升山区6县职教中心；完成学前教育三年行动计划，抓好50所公办示范性幼儿园新改扩建工程；加快推进15中整体迁建、24中整体改造、2中二期工程、中小学实践基地等教育设施建设；扩充优质教育资源，提升高中教育质量；加快发展职业教育、特殊教育、高等教育。深化群众性精神文明创建活动，大力提高市民素质和社会文明程度。提高公共文化设施服务水平，加快霞光大剧院（演艺中心）、奚啸伯大剧院等文化项目建设。完善公共文化服务网络，重点抓好市、县、乡、村四级文化阵地建设。广泛开展“文化三下乡”活动，继续办好“彩色周末”、“燕赵讲坛”等群众喜闻乐见的文化活动。抓好长城梦世界动漫城等一批重大文化产业项目。繁荣文化精品创作，提高我市品牌文化的知名度和美誉度。

提高就业和城乡居民收入水平。制定城乡统一的就业扶持政策，完善以就业为导向的职业培训体系；提升中低收入群体的收入水平，重点帮扶城镇就业困难人员、农村剩余劳动力和进城务工人员、退伍军人、高校毕业生等实现稳定就业。年内新增城镇就业9.9万人，农村劳动力转移就业5.7万人。

提高社会保障能力和人民群众健康水平。认真落实城镇企业职工基本养老保险政策，切实做好城中村居民参保工作。努力扩大医疗保险覆盖面，城镇职工、居民医保参保人数达到271万人，新农合参合率稳定在97%以上。积极开展城乡居民大病保险市级统筹试点。加快实施市级优质卫生资源倍增工程，扎实推进乡村卫生服务一体化管理，提高社区和乡村两级卫生机构的服务能力和服务质量。深化县级公立医院综合改革。抓好“国家中医药发展综合改革试验市”建设，提升基层中医药服务能力。引导和鼓励民间资本兴办养老机构，重点推进敬老院、光荣院、福利院“三院合一”建设。启动实施重度残疾人护理补贴制度，完成市残疾人康复中心改扩建项目建设。坚持计划生育基本国策，完善计生利益导向政策，切实做好计生特殊家庭的关怀扶助工作，提高计生家庭的社会保障水平。继续实施新生儿出生缺陷干预工程，提高出生人口素质。加快全民健身中心建设，打造城区“10分钟体育健身圈”。

切实加强食品药品管理。全面加强食品药品安全监管体系建设，完善市场监管信息化网络，提高监管技术支撑能力。及时公布食品药品安全信息。严格落实食品药品生产经营企业的主体责任和政府部门的监管责任。加大执法监督和查处力度，始终保持高压态势，严厉打击非法生产、销售行为，保障人民饮食用药安全。

加强和创新社会管理。扎实推进社会管理创新综合试点市建设，促进全市社会管理水平整体提升。加强基层组织和服务能力建设。进一步完善社会治安防控体系，严厉打击各类刑事犯罪。搞好流动人口、特殊人群的管理。健全公共安全应急机制，做好突发公共事件应急处置工作。严格落实安全生产责任制，坚决遏制重特大安全事故发生，进一步增强群众的安全感和满意度。推进网络依法规范有序运行。着力解决信访突出问题，依法维护信访秩序，保护妇女儿童合法权益，关心残疾人事业。积极支持驻石人民解放军和武警部队建设，大力加强国防教育和后备力量建设，认真落实优抚安置政策，持续巩固军政军民团结。支持工青妇等人民团体发挥桥梁纽带作用，切实做好防震减灾、民族宗教、外事侨务、气象、人防、社科、档案等各项工作，促进社会事业全面进步。

今年，我们要集中力量，继续办好利民惠民十件实事。提升供热质量。完成120个老旧小区供热二次管网改造，有效解决老旧小区供热不达标问题。缓解交通拥堵。实施11项道路改造和优化管理项目，完善智能化交通管理体系，重点对市区10个路口进行渠化改造，改造60处港湾式公交站台，建设3座人行过街天桥，打通4条断头路。改善空气质量。完成市区剩余的155台分散燃煤锅炉拆改工作，购置新型燃气公交车600辆，淘汰黄标车1万辆以上。加快保障性住房建设。新开工建设保障性住房及棚户区改造3.8万套，竣工2.6万套,分配入住2.2万套。整治老旧小区环境。对100个老旧小区道路、排水管网、路灯设施进行整修。认真解决老旧小区无人管理和脏乱差问题。增加食品便民网点。在市内社区建设50个惠民店。加快建设“放心馒头”二期工程，逐步满足市民需求。提高社区养老助残水平。在市内五区新建10个示范性社区居家养老服务中心、10个成年智力、精神、重度残疾人日间照料中心，对200户残疾家庭实施居家无障碍改造。解决农村饮水安全。新建联村集中供水工程20处，单村集中供水工程230处，解决390个村、60万人的饮水安全问题。加大医疗惠民力度。县级公立医院、政府办社区卫生服务机构，乡镇卫生院和村卫生室，全部实行基本药物零差率销售；启动新农合大病保险工作，基本解决因病返贫问题；扩大新农合重大疾病保障病种范围，保障病种由6类增加到20类。扩大再生资源回收。在全市农村建设350个再生资源固定回收站，建立9个县域再生资源集散中心，提高农村废旧物品利用率。

各位代表！人民对美好生活的向往，就是政府的奋斗目标。我们将始终把解决好人民群众最关心、最直接、最现实的利益问题，放在心上、抓在手上、落到实处，努力让全市人民过上美好生活!

四、努力建设人民满意的服务型政府

围绕建设创新政府、廉洁政府、法治政府的目标，进一步转变职能、改进作风、优化服务，全面提升政府工作效率和水平。

加快转变政府职能。把改革创新精神贯穿政府工作全过程，继续深化行政管理体制改革，推进政企分开、政资分开、政事分开、政社分开，加大向市场放权的力度，推进生产要素市场化，营造公平公正的市场环境。继续推进效能革命，真正把工作重心和主要精力转向创造良好发展环境、提供优质公共服务上来。按照权责一致的原则，调整部门职责权限，明确职责分工，进一步精简和规范行政审批事项，优化审批流程，力争在现有基础上再压缩30%。推进公共资源交易市场化改革，加快公共资源交易中心建设。加大绩效考核力度，全面提高办事效率和办事质量。提高行政服务中心规范化建设水平，加快推进网上行政服务中心建设，年内市本级所有适宜在网上运行的行政许可、非行政许可项目，实现网上办理和联合审批。创新行政管理方式，大力推进政府系统标准化建设。

全面推进依法行政。把遵守宪法和法律作为施政的根本原则，模范遵守和执行各项法律法规，严格按照法定权限行使权力，自觉接受人大及其常委会的法律监督和工作监督，自觉接受人民政协的民主监督，认真听取各民主党派、工商联、无党派人士和人民团体的意见。认真办理人大代表建议和政协提案。重视发挥新闻媒体和社会公众的监督作用，保障人民群众的知情权、参与权、表达权、监督权。加强重点领域政府立法，强化行政监察和审计监督。加强法制宣传教育，规范行政执法活动，严格落实行政执法责任制，真正做到有法必依、执法必严、违法必究。

切实改进工作作风。认真落实中央改进工作作风、密切联系群众八项规定和省委、市委的实施意见，大力弘扬西柏坡精神，牢记“空谈误国、实干兴邦”，坚决克服形式主义、官僚主义，坚持从市政府领导班子做起，要求别人做到的自己先做到，要求别人不做的自己坚决不做。大兴求真务实之风，带头深入基层，察实情、办实事、解难题，求实效，解决实际问题。大力改进文风会风，精简会议，减少文件，真正把精力用在抓落实上、把功夫下在见实效上。积极开展以“为民、务实、清廉”为主要内容的群众路线教育实践活动，时刻把群众的安危冷暖放在心上，多谋民生之利，多解民生之忧，多办民生之事，以实际行动践行全心全意为人民服务的根本宗旨。

大力加强廉政建设。全面贯彻“标本兼治、综合治理、惩防并举、注重预防”的方针，加强反腐倡廉教育和廉政文化建设，提高公务员队伍廉洁从政自觉性。厉行勤俭节约，力戒奢靡之风，严守公务接待要求，严格公务用车标准，严把文件会议质量，公务接待、公务出国、公务购车经费只减不增。深入开展民主评议活动，坚决纠正行业不正之风，认真解决群众反映强烈的热点、难点问题，切实维护人民群众的合法权益。加大案件查处力度，以反腐倡廉的实际成效取信于民。深化政务公开，让人民监督权力，让权力在阳光下运行，做到干部清正、政府清廉、政治清明，大力提高政府公信力。

各位代表！我们已经踏上转型升级、跨越赶超、建设幸福石家庄的新征程，我们肩负的责任重大而光荣。让我们紧密团结在以习近平同志为总书记的党中央周围，在省委、省政府和中共石家庄市委的正确领导下，紧紧依靠全市人民，以奋发有为的精神状态，务实高效的工作作风，扎实有力的工作举措，为率先全面建成小康社会而努力奋斗！
